# Epigallocatechin gallate protects mice from *Salmonella enterica* ser. Typhimurium infection by modulating bacterial virulence through quorum sensing inhibition

**DOI:** 10.3389/fcimb.2024.1432111

**Published:** 2024-10-16

**Authors:** Guoqiang Cheng, Shanqiu Jian, Wen Li, Liangchun Yan, Tiezhu Chen, Tingting Cheng, Zongxiu Liu, Gang Ye, Huaqiao Tang, Li Zhang

**Affiliations:** ^1^ Animal Experiment Center, Sichuan Academy of Chinese Medicine Sciences, Chengdu, China; ^2^ College of Veterinary Medicine, Sichuan Agricultural University, Chengdu, China; ^3^ Department of Science, Sichuan Academy of Agricultural Sciences, Chengdu, China; ^4^ Department of Innovation, Chengdu Qiankun Animal Pharmaceutical Co., Ltd, Chengdu, China

**Keywords:** epigallocatechin gallate, *Salmonella enterica* ser. Typhimurium, quorum sensing, type III secretion system, virulence

## Abstract

*Salmonella enterica* ser. Typhimurium is a common pathogen that poses a considerable public health threat, contributing to severe gastrointestinal diseases and widespread foodborne illnesses. The virulence of *S.* Typhimurium is regulated by quorum sensing (QS) and the type III secretion system (T3SS). This study investigated the inhibitory effects and anti-QS activity of epigallocatechin gallate (EGCG), which is a bioactive ingredient found in green tea, on the virulence of *S.* Typhimurium. *In vitro* bacterial experiments demonstrated that EGCG inhibited the production of autoinducers, biofilm formation, and flagellar activity by downregulating the expression of AI-1, AI-2, *Salmonella* pathogenicity islands (SPI)-1, SPI-2, and genes related to flagella, fimbriae, and curli fibers. In a mouse model of *S.* Typhimurium-induced enteritis, EGCG considerably reduced intestinal colonization by *S*. Typhimurium and alleviated intestinal damage. In conclusion, EGCG protects the intestines of mice infected with *S.* Typhimurium by inhibiting QS-induced virulence gene expression, demonstrating its potential as a therapeutic agent for controlling *S.* Typhimurium infections.

## 1 Introduction

Epigallocatechin gallate (EGCG), which is the most abundant catechin in green tea, is a flavonoid with eight hydroxyl groups, playing a crucial role in oxidative stress, cardiovascular diseases, viral infections, inflammation, and therapeutic potential in cancer and some bacterial diseases (Ramírez-Mares and de Mejía, 2003; Keller and Wallace, 2021; Wu et al., 2021; Zhang et al., 2021). Additionally, EGCG exhibits inhibitory effects on the growth of gram-positive and -negative bacteria, such as *Staphylococcus aureus* (Novy et al., 2013), *Escherichia coli* (Bo et al., 2023), *Acinetobacter baumannii* (Yan et al., 2021), *Pseudomonas aeruginosa* (Hao et al., 2021), and *Salmonella enterica* ser. Typhimurium (Chang et al., 2020). *S.* Typhimurium is a common pathogen of foodborne gastroenteritis and is primarily transmitted through the ingestion of contaminated water or food. In addition to affecting humans, *S*. Typhimurium infects domestic animals, including cattle, pigs, and poultry (Scallan et al., 2011; Chong et al., 2021). The emergence and spread of multidrug-resistant bacteria, such as *S*. Typhimurium, pose a severe threat to human and animal health globally (Shi et al., 2022). The escalating issues of bacterial antibiotic resistance impede the clinical application of antibiotics, rendering the development of non-bactericidal antivirulence agents an urgent priority.

Quorum sensing (QS) is a cell–cell communication process regulated by autoinducers (AIs) (Stenvang et al., 2016). When a bacterial population reaches a critical density, the accumulation of AIs surpasses a threshold, triggering a signaling cascade that synchronizes the expression of genes involved in bioluminescence, virulence, biofilm formation, and other biological processes (Cox et al., 2016; Chen et al., 2019; Nahar et al., 2021; Dawan et al., 2022). *S*. Typhimurium possesses at least two QS systems: one regulated by AI-1 and the other by AI-2. Although *S*. Typhimurium can detect exogenous acyl-homoserine lactones (AHLs) through LuxR family receptors, it does not produce AHLs endogenously (Dyszel et al., 2010). *S*. Typhimurium processes a fully functional AI-2 signaling system encoded by the luxS synthase and *lsrR* receptor adjacent to the *lsrACDBFG* operon (Elfaky et al., 2022). Cell invasion relies on the species-specific type III secretion system (T3SS), which is associated with *Salmonella* pathogenicity islands 1 (SPI-1) and 2 (SPI-2) (Ochman et al., 1996; Shea et al., 1996). SPI-1 is crucial for host cell invasion (Lou et al., 2019), whereas SPI-2 predominantly facilitates the intracellular survival of *S*. Typhimurium (Fass and Groisman, 2009). luxS-mediated QS is essential for the regular expression of specific SPI-1 genes, and the absence of the *luxS* gene reduces SPI-1 transcription, thereby attenuating the virulence of *Salmonella* species (Choi et al., 2012). In suitable environments, the mutual activation of *hilD*, *hilC*, and *rtsA* amplifies environmental signals, acting as a switch for full SPI-1 activation and leading to epithelial invasion (Chowdhury et al., 2021). Within SPI-2, the *ssrA* and *ssrB* genes encode the SsrA–SsrB two-component system, where SsrA is the sensor kinase and SsrB is the response regulator. SsrB directly induces the expression of SPI-2 genes and other virulence genes outside SPI-2 (Banda et al., 2019). *S*. Typhimurium utilizes specific QS and T3SS mechanisms to modulate its pathogenicity. A rapid increase in highly transmissible multidrug-resistant *S*. Typhimurium strains necessitates the development of safe and effective treatments. The synergistic effects of EGCG combined with antibiotics have been extensively examined (Luiz de Freitas et al., 2021), but the potential of EGCG as a QS inhibitor for reducing bacterial virulence remains underexplored. In this study, we evaluated the inhibitory effects of EGCG on AI-1 and AI-2 production, motility, and biofilm formation of *S*. Typhimurium by examining its effects on *S*. Typhimurium virulence gene expression and *S*. Typhimurium-induced enteritis in mice. The results revealed that EGCG considerably suppressed the production of key virulence factors without compromising pathogen growth, demonstrating that EGCG is a promising alternative treatment for controlling *S*. Typhimurium infection in animals.

## 2 Materials and methods

### 2.1 Bacterial strain, growth conditions, and reagents

#### 2.1.1 Determination of minimum inhibitory concentration and bacterial growth

*S*. Typhimurium was cultivated on a TSA medium, and a single colony was then inoculated and cultured in a TSB medium. The minimum inhibitory concentration (MIC) of EGCG for *S*. Typhimurium was determined through microtiter broth dilution. The OD_600_ value of the bacterial suspension as the work solution was briefly adjusted to 6 × 10^−4^ with a TSB medium. According to the formula we established (Y = 15.93X × 10^8^), 9.558 × 10^5^ CFU *S*. Typhimurium was present in the work solution. Then, the work solution was distributed to a 96-well plate (195 μL per hole). An EGCG solution (5 μL) was transferred to the first hole, and the concentration was 2,048 μg/mL. The drug was diluted to a concentration of 64 μg/mL. After 24 h of culture, the clear hole would be selected as the MIC. For the determination of bacterial growth, an *S*. Typhimurium work solution was treated with different concentrations of EGCG (0 μg/mL–400 μg/mL) at 37°C by shaking at 160 rpm. The OD_600_ value was recorded every hour with a spectrophotometer, and a growth curve was plotted. The experimental group without EGCG was set as the DMSO control group in this section and the following experiments.

#### 2.1.2 AI-1 and AI-2 detection

AI-1 was detected using previously described methods (Luiz de Freitas et al., 2021). An overnight culture of *Chromobacterium violaceum* 12472 was briefly diluted in a 1:100 ratio with 10 mL of molten agar. After solidification, various concentrations of EGCG (0 μg/mL, 100 μg/mL, 200 μg/mL, and 400 μg/mL) were added to Petri dishes and incubated at 37°C for 24 h. The Petri dishes were then observed for bacterial growth and color changes.

SAI-2 was detected using a bioluminescence assay with minor modifications (Gao et al., 2016). Briefly, *S*. Typhimurium was cultured overnight with various concentrations of EGCG. The culture suspension was centrifuged at 12,000 *g* for 10 min, and the supernatant was collected and filtered through a 0.22-μm filter. The reporter strain *Vibrio harveyi* BB152 (positive control) was cultured overnight at 30°C in an AB medium, and then cell-free supernatants were collected with a microporous filter membrane (0.22 μm). *V. harveyi BB170* was cultured at 30°C for 18 h in an AB medium and diluted (1:5,000) with a fresh AB medium. Diluted cells (180 μL) were added to a 96-well plate and mixed with 20 μL of cell-free supernatant containing either *S*. Typhimurium or *V. harveyi* BB152. The bioluminescence intensity within 0.5 s was detected using a multifunctional microplate reader after 3 h of incubation. The assay was repeated three times. AI-2 activity was quantified at a relative AI-2 concentration with the following formula: $\text{Relative AI}-2\text{ concentration}=\frac{\text{Nt}}{{\text{N}}_{\text{P}}}\times 100\%$ , where Nt is the experimental sample’s bioluminescence and Np is the bioluminescence of the positive control.

#### 2.1.3 Biofilm inhibition assay

A biofilm inhibition assay was performed using previously described methods with slight modifications (Balakrishnan et al., 2020). Briefly, an *S*. Typhimurium work solution was added to a 96-well plate, and varying concentrations of EGCG (0 μg/mL, 100 μg/mL, 200 μg/mL, and 400 μg/mL) were set. Then, the plate was incubated at 37°C for 48 h. After incubation, the culture medium was removed, and the plates were carefully washed three times with PBS. After the biofilm was fixed with 100 μL of methanol, 200 μL of 0.5% crystal violet was added, and unbound stain was rinsed with water. Residual water was removed by inverting the plate on a filter paper, and the plate was dried in an oven at 37°C. Biofilm bound to crystal violet was solubilized with 200 μL of 70% glacial acetic acid. The absorbance was measured at 595 nm with a microplate reader.

For an intuitive assessment of the effect of EGCG on *S*. Typhimurium biofilm formation, bacterial cultures were mixed with different concentrations of EGCG on a slide. Subsequently, the slide was washed multiple times with PBS, immersed in 2.5% glutaraldehyde for at least 24 h, dried, and dehydrated with 30%, 50%, 70%, 90%, and 100% ethanol. The dried sample was sprayed with gold and observed under a scanning electron microscope (ZEISS Sigma 300, Germany).

#### 2.1.4 Transmission electron microscopy

The effect of EGCG on *S*. Typhimurium flagella was explored, and the purified flagellar structures of *S*. Typhimurium and shapes and size were examined through transmission electron microscopy (TEM). In brief, *S*. Typhimurium work solution was cocultured with different EGCG (0 μg/mL, 100 μg/mL, 200 μg/mL, and 400 μg/mL) concentrations for 12 h and centrifuged at 8,000 g for 5 min. After removing the supernatant, the samples were fixed in 4% glutaraldehyde for 24 h, resuspended by vertexing, and deposited onto custom-made copper grids for observation (ht7800, Japan). Different fields of view were selected for observation and comparison, and the photographs of typical changes were obtained.

#### 2.1.5 Swimming motility assay

The swimming motility assay was conducted using previous methods with minor modifications (Abbas et al., 2020). *S*. Typhimurium was cultured overnight until its OD_600_ reached 0.7 and then inoculated into swimming agar plates containing agar (0.3%), peptone (1%), glucose (1%), and NaCl (0.5%) with 100 μg/mL, 200 μg/mL, and 400 μg/mL EGCG or without EGCG. The plates were incubated at 37°C for 12 h, and the diffuse colony zones were measured.

#### 2.1.6 Animal experiment

All animal experiments were conducted under the guidance of the animal welfare and ethics committee of Sichuan Agricultural University (Approval No. 20230045). ICR mice (18 g–20 g, 6 weeks) purchased from SPF Biotechnology Co., Ltd. (Beijing, China), were housed in a controlled environment (12-h light–12-h dark cycle at room temperature [23°C ± 1°C]) with free access to food and water. After acclimatization, the mice were randomly assigned to five groups (n = 10 per group): control (saline), model (*S*. Typhimurium), and EGCG (80 mg/kg, 40 mg/kg, or 20 mg/kg) + *S*. Typhimurium. The mice in the ECGC + *S*. Typhimurium groups were orally pretreated with EGCG for 7 days once daily before *S*. Typhimurium infection, whereas the mice in the model group were infected without pretreatment. Before infection, the mice were pretreated with 100 μL of 5% NaHCO_3_ solution to neutralize gastric acid and received 100 μL of *S*. Typhimurium suspension (10^9.5^ CFU/mL) through oral gavage on days 4 and 6 of drug treatment. Animal body weight was recorded, and change in weight was calculated by comparing the final weight with the initial weight before *S*. Typhimurium infection. Upon the completion of the experiment, the mice were anesthetized with tribromoethanol (1.25%) and sacrificed, and the spleen/body weight ratio was calculated. The colon was separated, measured by length, and fixed in 10% formaldehyde for 2 days. The colonic tissue blocks were dehydrated, embedded, and prepared into 3-μm sections, which were observed and photographed under a light microscope after hematoxylin–eosin staining. After the intestinal contents were carefully removed, the intestinal mucosa was scraped, and a certain proportion of sterile PBS was collected for homogenization. The suspension was then spread on a *Salmonella* selective medium (Qingdao Haibo Biotechnology Co., Ltd., Qingdao) and counted after culture.

#### 2.1.7 Statistical analysis

All reported experiments were independently repeated at least three times. Statistical analysis was carried out in SPSS 20.0 (IBM, USA), and results are expressed as mean ± standard deviation ( $\bar{x}$ ± SD). The data were compared among the groups using one-way analysis of variance and Tukey’s *post-hoc* analysis.

## 3 Results

### 3.1 Effect of EGCG on bacterial growth

**Figure 1:**
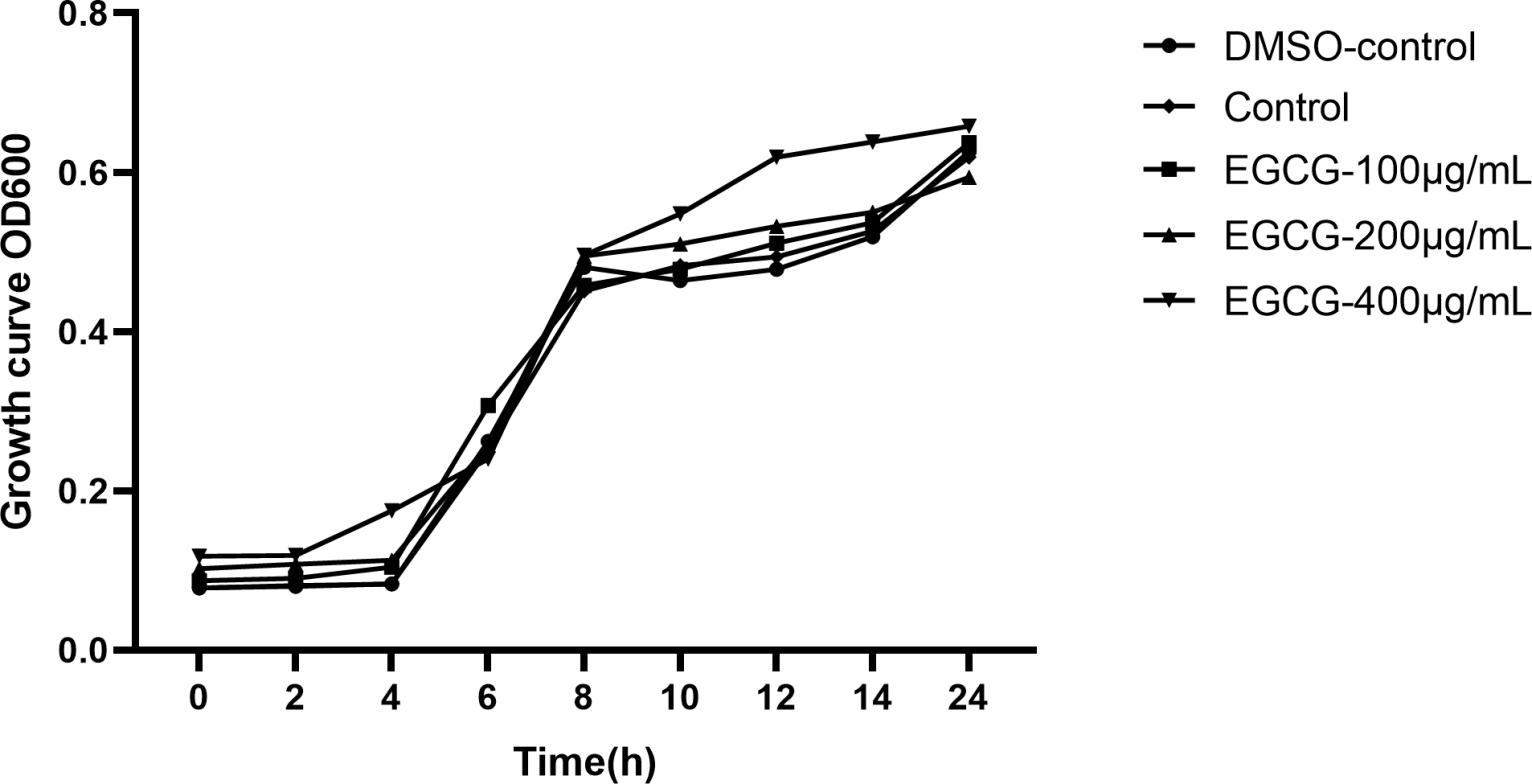
Growth curve of *S.* Typhimurium.

The MIC of EGCG for *S*. Typhimurium was 1,024 μg/mL. Subsequent experiments were conducted at sub-MICs (400 μg/mL, 200 μg/mL, and 100 μg/mL) to determine the inhibitory effect of ECGC on *S*. Typhimurium growth. The growth curves indicated that DMSO and these sub-MICs of EGCG had no considerable effect on the growth of *S*. Typhimurium (**Figure 1**).

### 3.2 Effect of EGCG on AI-1 and AI-2 production

**Figure 2:**
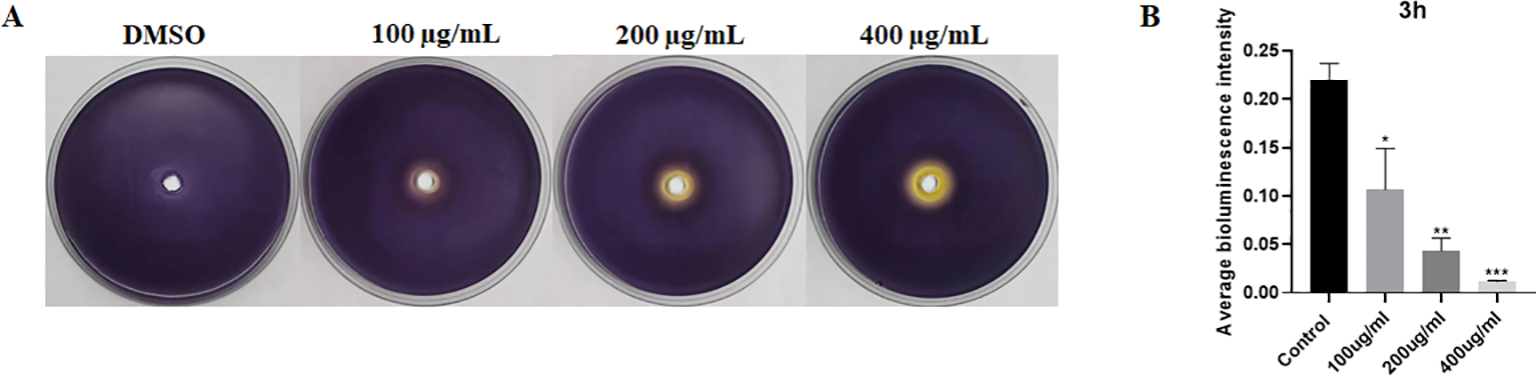
ECGC inhibited AI-1 and AI-2 production. **(A)** Visual qualitative assay for inhibition of *C. violaceum* violacein. **(B)** Measurement of AI-2 activity using a bioluminescence assay. All data are representative of three independent experiments performed in triplicates and are expressed as mean ± SD. *p < 0.05; **p < 0.01; ***p < 0.001.

To evaluate the influence of EGCG on AI molecule production, we performed purple pigment and bioluminescence analyses to detect the levels of AI-1 and AI-2. As shown in **Figure 2A**, EGCG inhibited violacein production compared with control. In addition, EGCG inhibited AI-2 secretion by *S*. Typhimurium compared with the control in a concentration-dependent manner (**Figure 2B**). The results demonstrated that EGCG treatment inhibited the production of AI-1 and AI-2.

### 3.3 Effect of EGCG on biofilm formation

**Figure 3:**
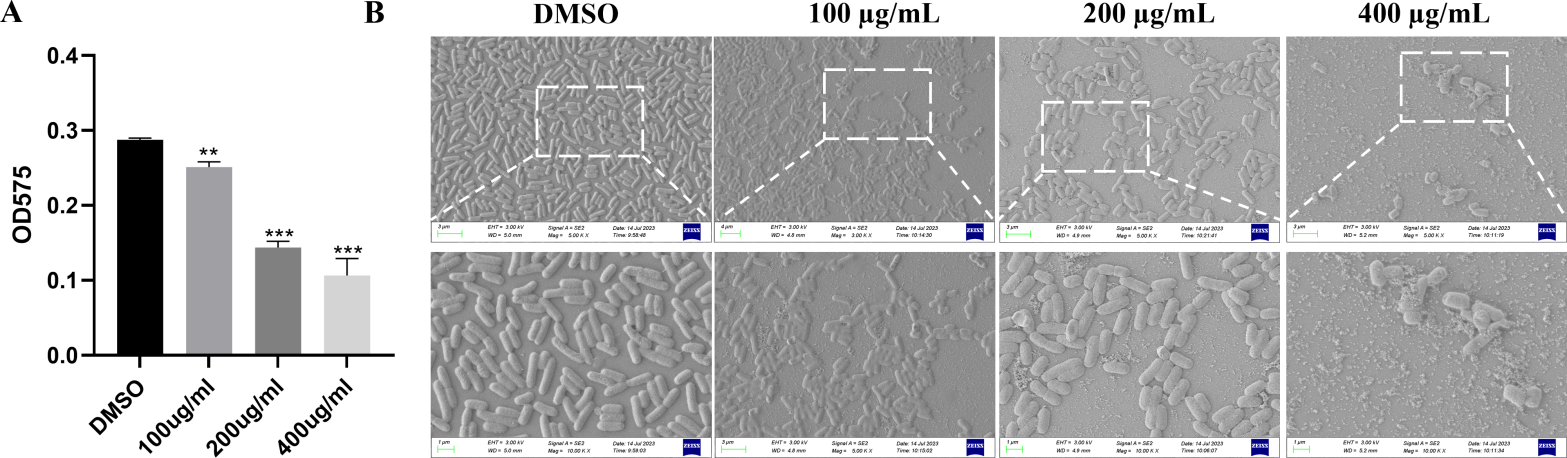
ECGC inhibited the biofilm formation. **(A)** Effect of 48-h EGCG treatment on biofilm formation. **(B)** SEM of biofilm. All data are representative of three independent experiments performed in triplicates and are expressed as mean ± SD. **p < 0.01; ***p < 0.001.

The crystal violet staining method and scanning electron microscopy (SEM) were used in evaluating the influence of EGCG on biofilm formation to determine the product of biofilm. As shown in **Figure 3A**, EGCG considerably inhibited the formation of *S*. Typhimurium biofilms in a concentration-dependent manner. The biofilm inhibition rates of 400 μg/mL, 200 μg/mL, and 100 μg/mL EGCG were 63.3%, 50.0%, and 12.6%, respectively. For the visual assessment of biofilm growth, *S*. Typhimurium was cultured on glass slides overnight with different concentrations of EGCG and observed through SEM. EGCG treatment inhibited *S*. Typhimurium biofilm formation (**Figure 3B**), which was consistent with the result of biofilm formation.

### 3.4 EGCG inhibited flagellum formation

**Figure 4:**
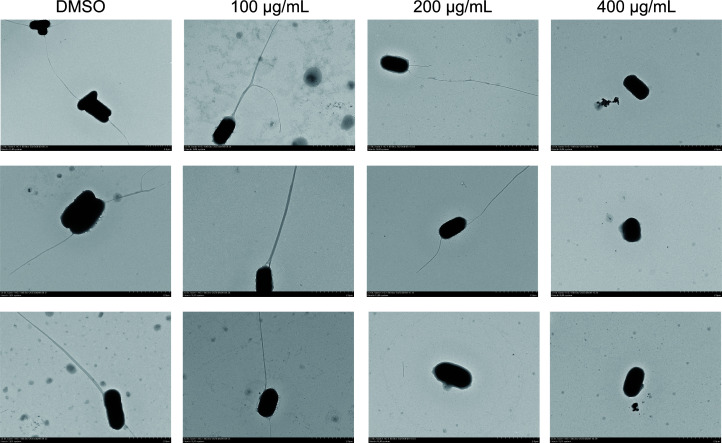
TEM images of *S.* Typhimurium flagellum (5,000× magnification).

The bacterial flagellum is a highly complex molecular structure that plays a vital role in pathogenesis, enabling bacterial to reach optimal host sites and facilitating colonization or invasion, persistence at infection sites, and postinfection dissemination (Chaban et al., 2015). The untreated *S*. Typhimurium in our study had intact flagella, whereas the EGCG-treated bacteria had shortened or fragmented flagella (**Figure 4**). Furthermore, flagella were absent in *S*. Typhimurium treated with 400 μg/mL EGCG.

### 3.5 EGCG impaired the swimming motility of *S*. Typhimurium

**Figure 5:**
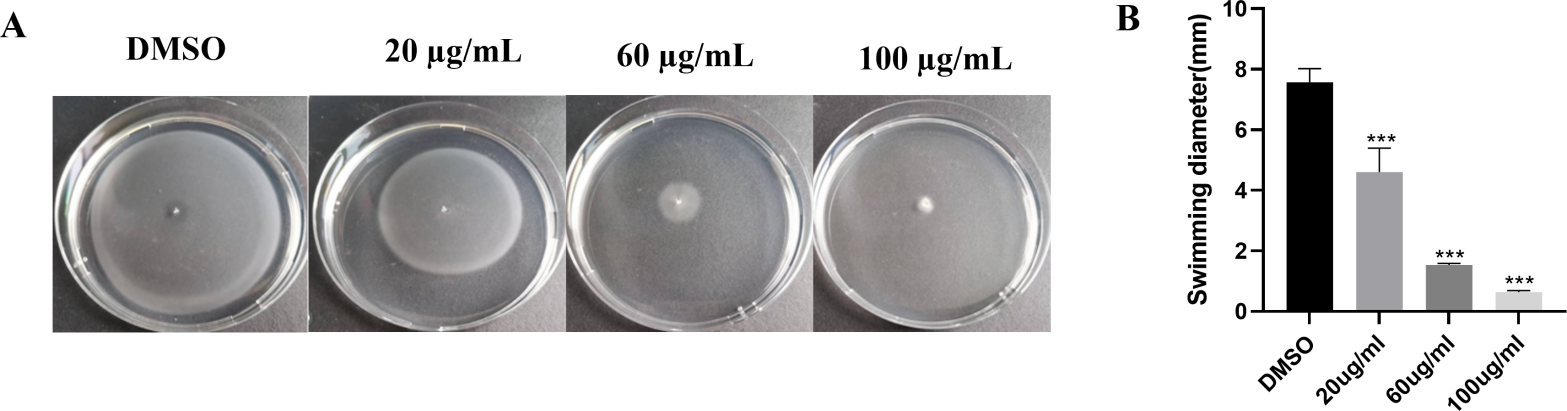
EGCG inhibited the swimming motility of *S.* Typhimurium. **(A)** Swimming inhibition rate of EGCG; **(B)** swimming distance of *S.* Typhimurium. All data are representative of three independent experiments performed in triplicates and are expressed as mean ± SD. ***p < 0.001.

The swimming motility assay revealed that EGCG inhibited the swimming motility of *S*. Typhimurium in a concentration-dependent manner (**Figure 5**). The swimming inhibition rates of 20 µg/mL, 60 µg/mL, and 100 µg/mL EGCG were 39.20%, 79.73%, and 91.62%, respectively. When the EGCG concentration exceeded 100 µg/mL, *S*. Typhimurium failed to swim toward the agar periphery.

### 3.6 Effect of EGCG on gene expression

**Figure 6:**
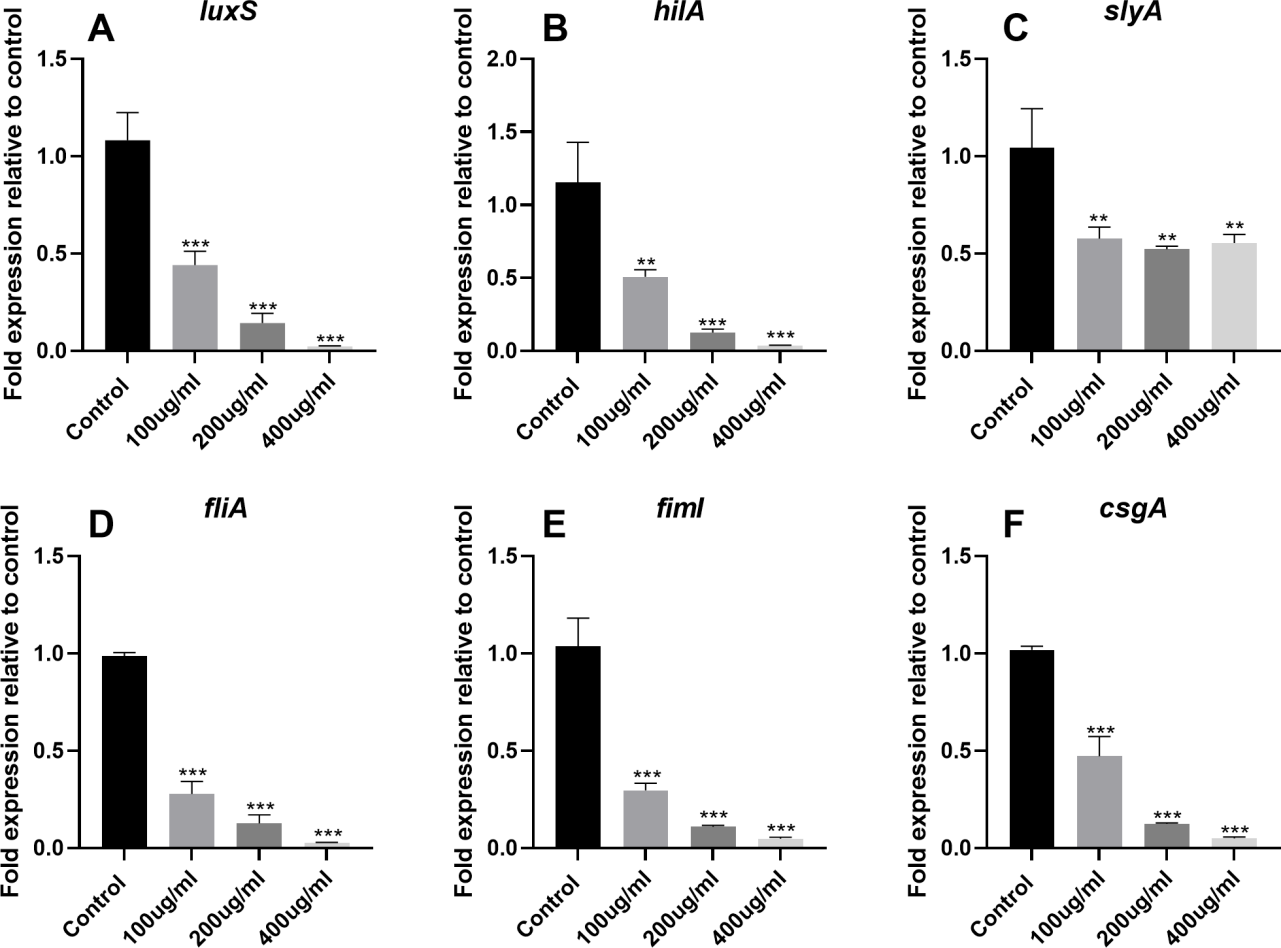
Effects of EGCG on QS, SPI-1, SPI-2, flagella, fimbriae, and curli fiber gene expression in *S.* Typhimurium. The gene expression of **(A)** LuxS, **(B)** hilA, **(C)** slyA, **(D)** fliA, **(E)** fiml, and **(F)** csgA. All data are representative of three independent experiments performed in triplicates and are expressed as mean ± SD. **p < 0.01; ***p < 0.001.

To further evaluate the anti-QS activity of EGCG, we assessed the expression of AI-1 (*sdiA*, *srgC*, and *pefI*) and AI-2 (*luxS*) genes. qPCR analysis revealed that EGCG significantly downregulated the expression of AI-1 and AI-2 genes in *S*. Typhimurium, which was in line with our findings from the violacein and bioluminescence assays. In addition, EGCG significantly inhibited the expression of SPI-1 (*hilC, hilE, hilD*, and *hilA*) and SPI-2 (*slyA, ssrA*, and *ssrB*) genes (p<0.01). Moreover, EGCG suppressed the expression of virulence factors associated with QS and T3SS, including genes related to flagella (*flgM, fliA, flhD*, and *fliZ*), fimbriae (*fimD, fimI, fimH, fimY*, and *fimZ*), and curli fibers (*csgE, csgB, csgG, csgA*, and *csgD*; **Figure 6** and **Supplementary Figure S1**).

### 3.7 ECGC attenuated *S*. Typhimurium infection in mice

**Figure 7:**
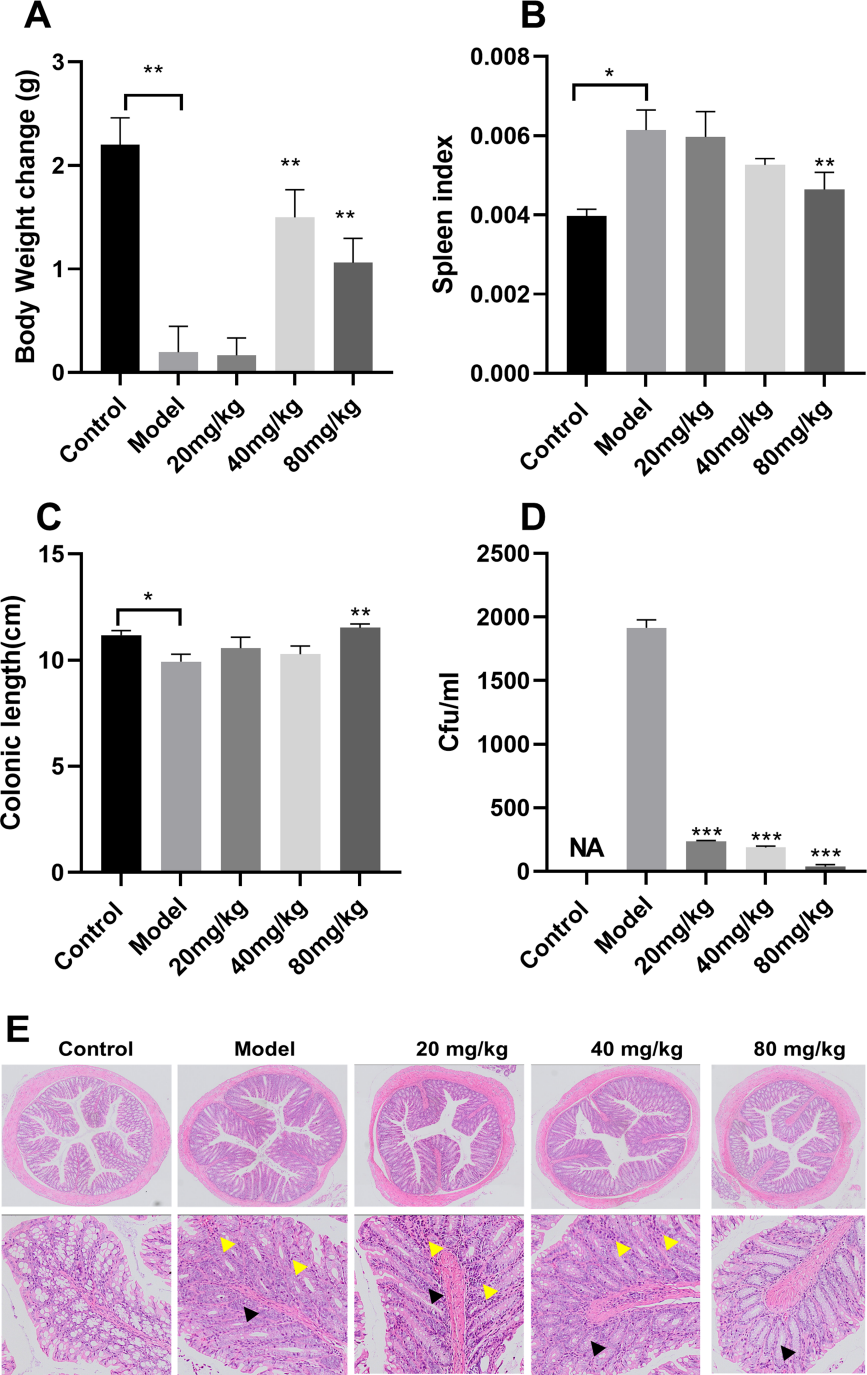
ECGC attenuated *S.* Typhimurium infection in mice. **(A)** Body weight of each group. **(B)** Spleen index of each group. **(C)** Colon length of each group. **(D)** Bacterial load of each group. **(E)** Colon pathology examination (decreased number of goblet cells ▲ and Inflammatory cell 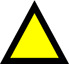 infiltration). All data are representative of three independent experiments performed in triplicates and are expressed as mean ± SD. *p < 0.05; **p < 0.01; ***p < 0.001.

EGCG treatment resulted in a considerable larger weight gain in the 40-mg/kg group than in the model group (**Figure 7A**). The spleen index was significantly higher in the model group than in the control group and lower in the EGCG group than in the model group (**Figure 7B**). In addition, the colon was considerably longer in the mice treated with high concentrations of EGCG than in untreated mice (**Figure 7C**). The effect of EGCG on bacterial adhesion was determined using the adherent *S*. Typhimurium recovered from the colons of the infected mice. EGCG prevented *S*. Typhimurium adhesion to the intestinal epithelium (**Figure 7D**). Compared with the model group, the 20-mg/kg, 40-mg/kg, and 80-mg/kg EGCG treatment groups exhibited 87.65%, 90.12%, and 98.07% decreases in bacterial CFU, respectively. Colon pathology revealed decreased goblet cells and glands and infiltration of inflammatory cells in the submucosa after *S*. Typhimurium infection (**Figure 7E**), but EGCG treatment alleviated these pathological changes in the colon.

## 4 Discussion

*S*. Typhimurium is a pathogen that causes a wide range of diseases, including gastroenteritis and systemic infections, affecting various hosts, especially humans (Herrero-Fresno and Olsen, 2018). Owing to the widespread use of antibiotics in clinical practice and livestock production, *S*. Typhimurium has developed resistance to multiple antibiotics, such as β-lactams, aminoglycosides, sulfonamides, tetracyclines, and quinolones (Li et al., 2024). Therefore, novel antimicrobial agents are essential to the effective management of these multidrug-resistant strains. Targeting bacterial virulence has emerged as a promising strategy for reducing pathogenicity without promoting antibiotic resistance, thereby effectively mitigating the spread and harmful effects of bacterial infections (Hegazy et al., 2022). Anti-QS agents can greatly reduce selective pressure on pathogens and delay the onset of drug resistance, demonstrating their effectiveness for antivirulence therapy (Tang and Zhang, 2014). Several studies have investigated the effects of QS-targeted natural products. AHL (AI-1) is primarily produced by gram-negative bacteria and facilitates intraspecies communication, which can be disrupted by various natural compounds, including paeonol (Yang et al., 2021), quercetin (Ren et al., 2024), and indole (Paopradit et al., 2022). EGCG downregulates AHL production and associated gene expression and thus inhibits biofilm formation; protease and elastase activity; and the swimming and swarming motility of *P. aeruginosa* (Hao et al., 2021). AI-2 is a common QS molecule produced by gram-negative and -positive bacteria (Scutera et al., 2014). Roy et al (Roy et al., 2022). demonstrated that quercetin inhibits biofilm formation, impairs swimming and swarming motility, and induces the cell lysis of *S*. Typhimurium by suppressing the expression of virulence and QS genes. In this study, EGCG inhibited the production of violacein in *C. violaceum* 026 and luminescence in *V*. *harvey*i BB152, suggesting that EGCG functions as a QS inhibitor by suppressing AI-1 and AI-2 production in *S*. Typhimurium. Consistent with our findings, the findings of Hosseinzadeh et al (Hosseinzadeh et al., 2020). demonstrated that EGCG exhibits anti-QS activity by downregulating the expression of AI-2-related genes (*sdiA* and *luxS*) in *S*. Typhimurium. Furthermore, our qPCR results confirmed that EGCG inhibited the expression of key QS regulators, including *sdiA, srgC, pefI*, and *luxS*. The pathogenesis of *Salmonella* is facilitated by the T3SS encoded by genes in SPI-1 and SPI-2 (Figueira and Holden, 2012; Lou et al., 2019). Moreover, EGCG attenuates the virulence of *E. coli, S*. *Typhimurium*, and *Yersinia pseudotuberculosis* by suppressing T3SS-related gene expression (Lun et al., 2017; Nakasone et al., 2017). Our qPCR data revealed that EGCG severely inhibited the expression of SPI-1 (*hilC, hilE, hilD*, and *hilA*) and SPI-2 genes (*slyA, ssrA*, and *ssrB*). However, the relationship between QS and SPI gene expression remains unclear. The QS system and T3SS are closely associated with the virulence of *S*. Typhimurium. To further validate the attenuation of *S*. Typhimurium virulence, we assessed the expression of genes related to flagella, pili, and curli. The *flhDC* operon, encoding the master regulatory proteins FlhD and FlhC, is positioned at the top of the flagellar transcriptional hierarchy (Guo et al., 2014). Two key classes of flagellar genes, namely, *fliA* and *flgM*, are regulated within this system. The flagellar regulon includes two critical regulatory factors (*flhDC* and *fliZ*), which serve as inducers of SPI-1 expression (Hung et al., 2012).

Mutations in *fliZ* considerably reduce *hilA* expression and impair intestinal colonization by *Salmonella* in mice (Chubiz et al., 2010). Our qPCR data indicated that EGCG treatment inhibited the expression of flagellum-related genes (*flgM*, *fliA*, *flhD*, and *fliZ*). This finding was supported by the TEM analysis results, which revealed flagellar fragmentation and loss after EGCG treatment. *fimI* and *fimH* encode the subunits of type 1 pili and are crucial for bacterial adhesion (De Buck et al., 2003; Lee and Yeh, 2016). Curli fibers, which are amyloid-like structures encoded by the *csgBAC* and *csgDEFG* operons, play a key role in biofilms generated by *S*. Typhimurium (Nicastro et al., 2019; Ou et al., 2023). In the present study, EGCG considerably downregulated the expression of fimbriae (*fimD, fimI, fimH, fimY*, and *fimZ*) and curli fiber genes (*csgE, csgB, csgG, csgA*, and *csgD*). Furthermore, crystal violet staining and SEM observations confirmed that EGCG treatment suppressed biofilm production by *S*. Typhimurium. The mouse model of *S*. Typhimurium infection provides a valuable tool for evaluating anti-intestinal inflammation drugs (Hapfelmeier and Hardt, 2005). Our experiments showed that EGCG preserved body weight and spleen index while reducing colon inflammation in *S*. Typhimurium-infected mice. Additionally, colon bacterial colonization by *S*. Typhimurium was considerably lower in the EGCG-treated mice, and histopathological analysis revealed the protective effect of EGCG against *S*. Typhimurium-induced enteritis. These findings suggest that EGCG is a promising and effective antibacterial agent for managing *S*. Typhimurium infections.

## 5 Conclusions

EGCG can attenuate the virulence of S. Typhimurium by inhibiting the QS, T3SS, and motility of the pathogen. Furthermore, EGCG exhibits protective effects against S. Typhimurium infection in mice. EGCG is a promising candidate for anti-virulence therapy as it targets QS and T3SS without imposing selective pressure on bacterial growth, which is crucial in widespread antibiotic resistance. Nonetheless, further studies are warranted to elucidate the specific mechanisms of EGCG in S. Typhimurium infection.

## Data Availability

The original contributions presented in the study are included in the article/**Supplementary Material**. Further inquiries can be directed to the corresponding authors.
