# Supplementary material for: Epigallocatechin gallate protects mice from Salmonella enterica ser. Typhimurium infection by modulating bacterial virulence through quorum sensing inhibition
Source: Front Cell Infect Microbiol. 2024 Oct 16;14:1432111. doi: 10.3389/fcimb.2024.1432111 (PMC11521958; doi:10.3389/fcimb.2024.1432111)

Supplementary file

Table S1 Primers of genes

| Target name | Type | Primer sequence |
| --- | --- | --- |
| LuxS | Fw | GCGACCACCTCAACGGTAACG |
|  | Rev | ACTTTCAGCACATCCGCCATCG |
| FilZ | Fw | CTTTAAACACAGCCAGACGCATTGC |
|  | Rev | CGATCTTGCCACGGCGAACC |
| sdiA | Fw | AGGAGATGGCGGCAGCAGAG |
|  | Rev | GGACGGGATGACGAACACACAG |
| srgC | Fw | GCATATCAGTGCGGGTACAGTAGTG |
|  | Rev | TTCCGTCAGGCTTGCATCTTGAAG |
| pdfI | Fw | ACTCAGTATCTACCAGGTCCGCTTG |
|  | Rev | ACTCCCTTTCCGGCATTCACTTTC |
| fimI | Fw | TAGCGGGGCTTGTTTTGATGTCG |
|  | Rev | CAGTTGTCCGCGCAGGTGAAC |
| fimD | Fw | AAACGCCCGTACCTGGACAAAC |
|  | Rev | CCATCCTGCCGCTGCTGTTG |
| fimH | Fw | GGCGGGGACAACGGTGAATTATAC |
|  | Rev | CCAGCGACACTATCGGTGATGC |
| fimZ | Fw | GGCACCGACGGCTTTACCTTAC |
|  | Rev | CGCCCGCTCTTATTGCTCTTCC |
| fimY | Fw | GTACCACGCAGGGAAAGACACC |
|  | Rev | CGGCAAGGCATAATTGAGCTGTTG |
| FlgM | Fw | ACCTTTGAAACCCGTTAGCACTGTC |
|  | Rev | CTGACGCCTGGCTGCATGAG |
| FlhD | Fw | GATCACCCGTTTGACTCAGGATTCG |
|  | Rev | CGCCGTATCGTCCACTTCATTGAG |
| FliA | Fw | TGTTGCGGAGTATCGTCAGATGTTG |
|  | Rev | CGCCCTCCAGCAGTTGATGTAAC |
| csgA | Fw | CAGCATTCGCAGCAATCGTAGTTTC |
|  | Rev | GCCGCCGCCGTTATGATTACC |
| csgB | Fw | TTGGTCAAGTCGGCACGGATAATAG |
|  | Rev | TCGCCCGATTATTTCCTCCTTCTTG |
| csgD | Fw | AATGCGGACTCGGTGCTGTTG |
|  | Rev | GTGGTCAGCGGATTACAGGGTATTC |
| csgE | Fw | GGTGTATTCGCTTTCCCATTTGTCG |
|  | Rev | CAGCAGAGTTACTGTTCGCTACCG |
| csgG | Fw | GCCTGTCCGCTTTATTCTGCAAATC |
|  | Rev | CTGGAAGGCGAAATCGGCTATACC |
| HilA | Fw | AGAACATGCGATTAAGGCGACAGAG |
|  | Rev | AGCAAACTCCCGACGATGTATTCTG |
| HilC | Fw | AAGCCCGGTGGGTTTGATTTCC |
|  | Rev | ACCTCAGCCTGTGACCATTTGC |
| HilE | Fw | TGGACGGTATTGAAGGCGAAAGC |
|  | Rev | GTTTTGTCGGGTTGTGGTTGTAAGC |
| HilD | Fw | TAACGTGACGCTTGAAGAGG |
|  | Rev | GGTAcCGCCATTTTGGTTTG |
| SlyA | Fw | CTAGGTTCTGATCTGGCACGGTTG |
|  | Rev | ACCCAATGTGTCTGCGTCAATTCC |
| SsrA | Fw | TTGCCGCAGAATACCAACTTTTACG |
|  | Rev | CAATATCAGCCAGCAAGAGGTCCAG |
| SsrB | Fw | CAGCAAGTTCTGTTAGCGGCATTG |
|  | Rev | AGCAGTTGATGATTGGTCGTGTCAG |

Fig S1 The gene expression of virulence


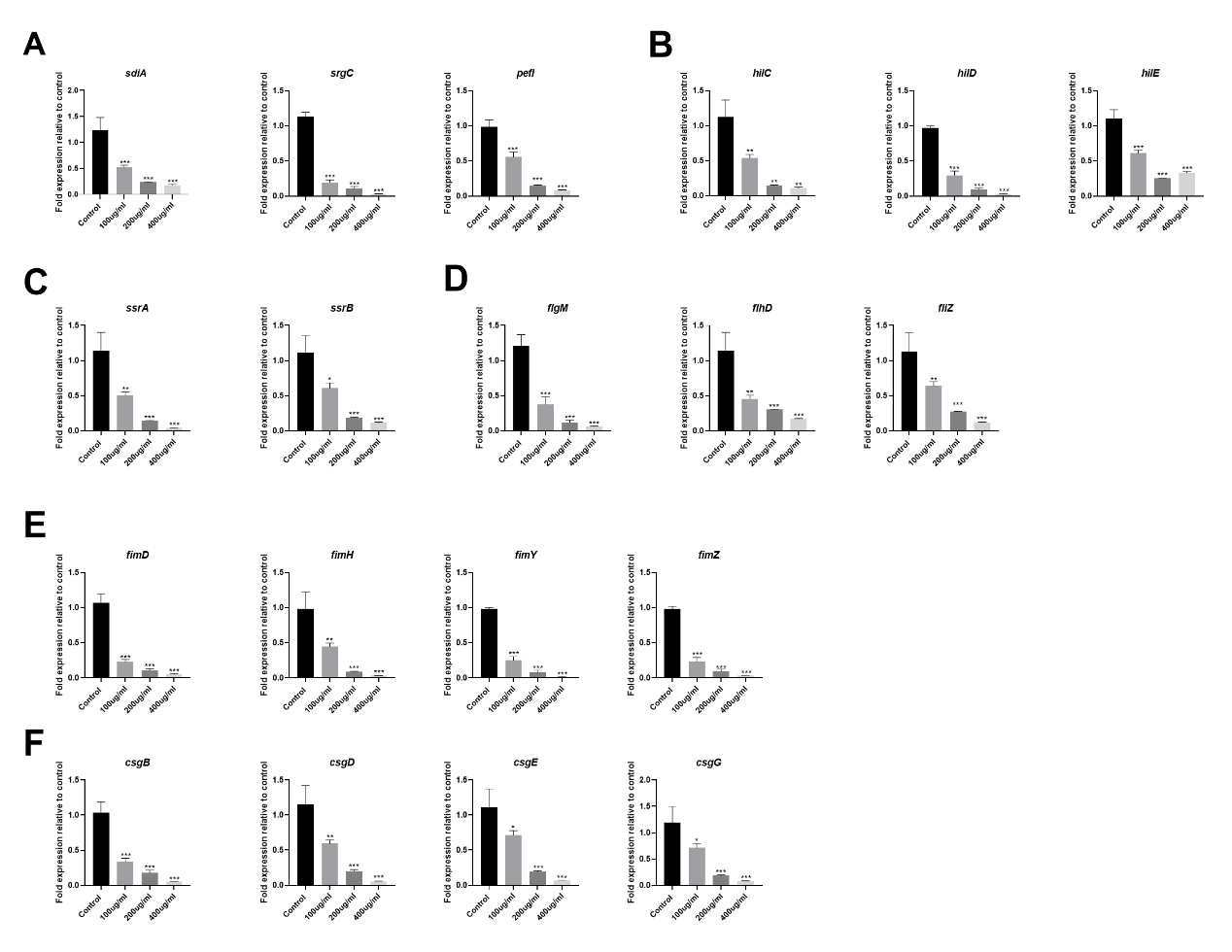

Supplement: Supplementary file 1 [file Table1.docx]
